# Supplementary material for: Bioprinted Cancer Model of Neuroblastoma in a Renal Microenvironment as an Efficiently Applicable Drug Testing Platform
Source: Int J Mol Sci. 2021 Dec 23;23(1):122. doi: 10.3390/ijms23010122 (PMC8745467; doi:10.3390/ijms23010122)
Supplement: Supplementary file 1 [file ijms-23-00122-s001.zip › ijms-1489485-supplementary.pdf]

Supplementary materials for

Bioprinted Cancer Model of Neuroblastoma Renal Metastasis as An Efficiently Applicable Drug Testing Platform

Dongwei Wu [1], Johanna Berg [1], Birte Arlt [2], Viola Röhrs [1], Munir A. Al-Zeer [1], Hedwig Deubzer [2,3,4,5] and Jens Kurreck [1,*]

[1] Institute of Biotechnology, Chair of Applied Biochemistry, Technische Universität Berlin, 13355 Berlin,
Germany; dongwei.wu@campus.tu-berlin.de (D.W.); johanna.berg@tu-berlin.de (J.B.); viola.roehrs@tu-berlin.de (V.R.); al-zeer@tu-berlin.de (M.A.A.-Z.)

[2] Department of Pediatric Hematology and Oncology, Charité-Universitätsmedizin Berlin, 13353 Berlin,
Germany; birte.arlt@charite.de (B.A.); hedwig.deubzer@charite.de (H.E.D.)

[3] Neuroblastoma Research Group, Experimental and Clinical Research Center (ECRC) of the Charité and the Max-Delbrück-Center for Molecular Medicine (MDC) in the Helmholtz Association, 13125 Berlin, Germany

[4] German Cancer Consortium (Deutsches Konsortium für Translationale Krebsforschung, DKTK), Partner Site Berlin, 10115 Berlin, Germany

[5] Berliner Institut für Gesundheitsforschung (BIH), 10178 Berlin, Germany

* Correspondence: jens.kurreck@tu-berlin.de; Tel.: +49-30-314-27-582; Fax: +49-30-314-27-502

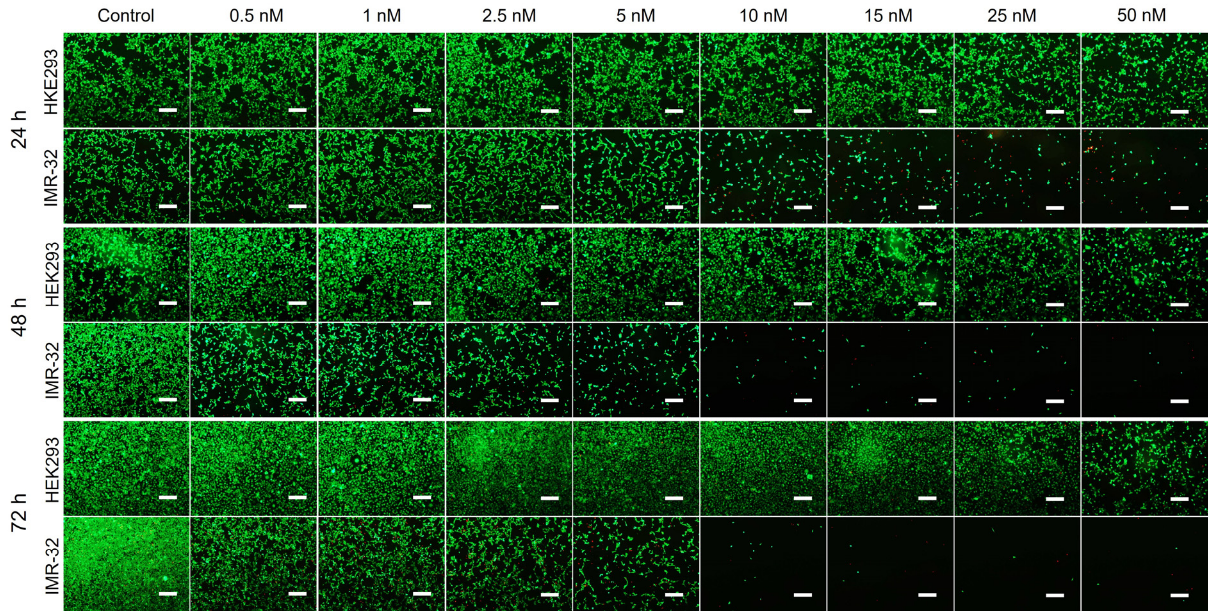

Figure S1. Cytotoxicity assay of 2D cultured HEK293 cells and IMR-32 cells after treatment with panobinostat. HEK293 cells and IMR-32 cells were cultured in monolayer and dosed with panobinostat at different concentrations (0-50 nM) for 24, 48 and 72 h. Living cells were stained in green while dead cells were stained in red, following image capture with a fluorescence microscopy. Scale bar, 250 μm.

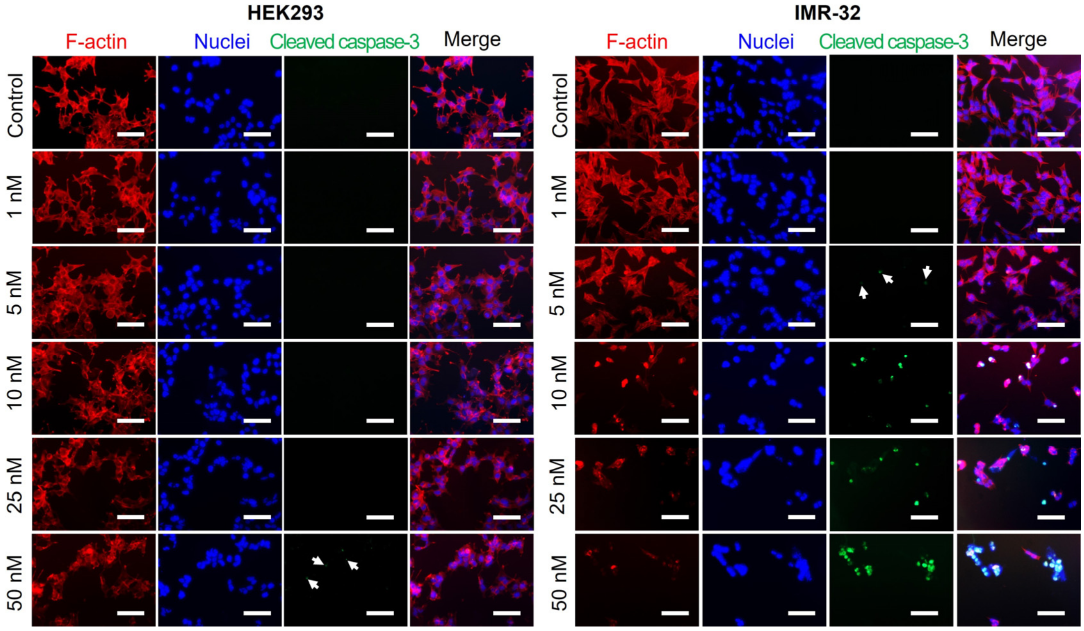

Figure S2. Induction of apoptosis in HEK293 and IMR-32 cells in response to panobinostat treatment visualized by immunofluorescence staining of cleaved caspase-3. Apoptotic cells were detected using cleaved caspase-3 antibody (green) 24 h post panobinostat treatment. F-actin and nuclei of all cells were stained with Alexa Fluor™ 594 phalloidin (red) and DAPI (blue), respectively. All images were taken at the same magnification; scale bar, 100 μm.

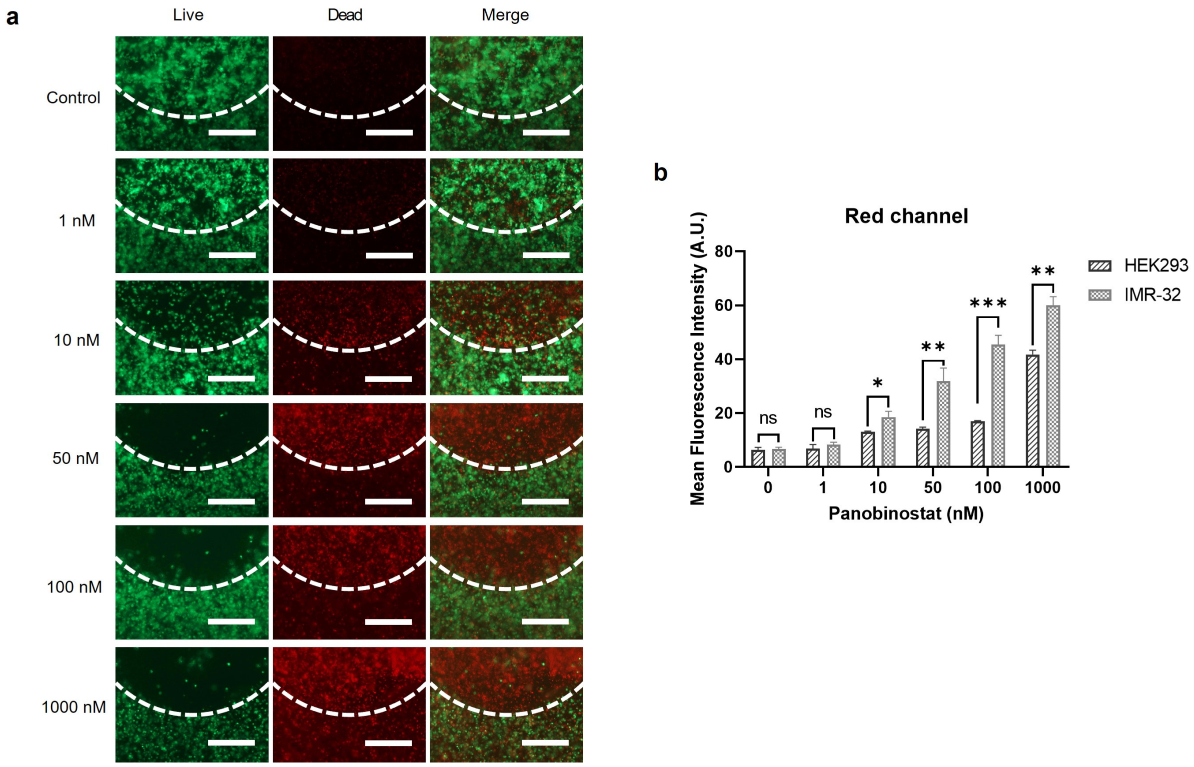

Figure S3. Cell viability of IMR-32 core and an environment of HEK293 cells in the bioprinted cancer models after treatment with panobinostat. (a) Cytotoxicity assays of cancer models were carried out after treatment with panobinostat for 72 h. Living cells appear in green, while dead cells are characterized by red fluorescence. The white dotted lines indicate the boundary of the cancer part (upper) and the non-cancerous environment (lower). Scale bar, 500 μm. (b) Quantitative analysis of dead cells in HEK293 and IMR-32 parts of the cancer models by mean fluorescence intensity based on the red channel results.

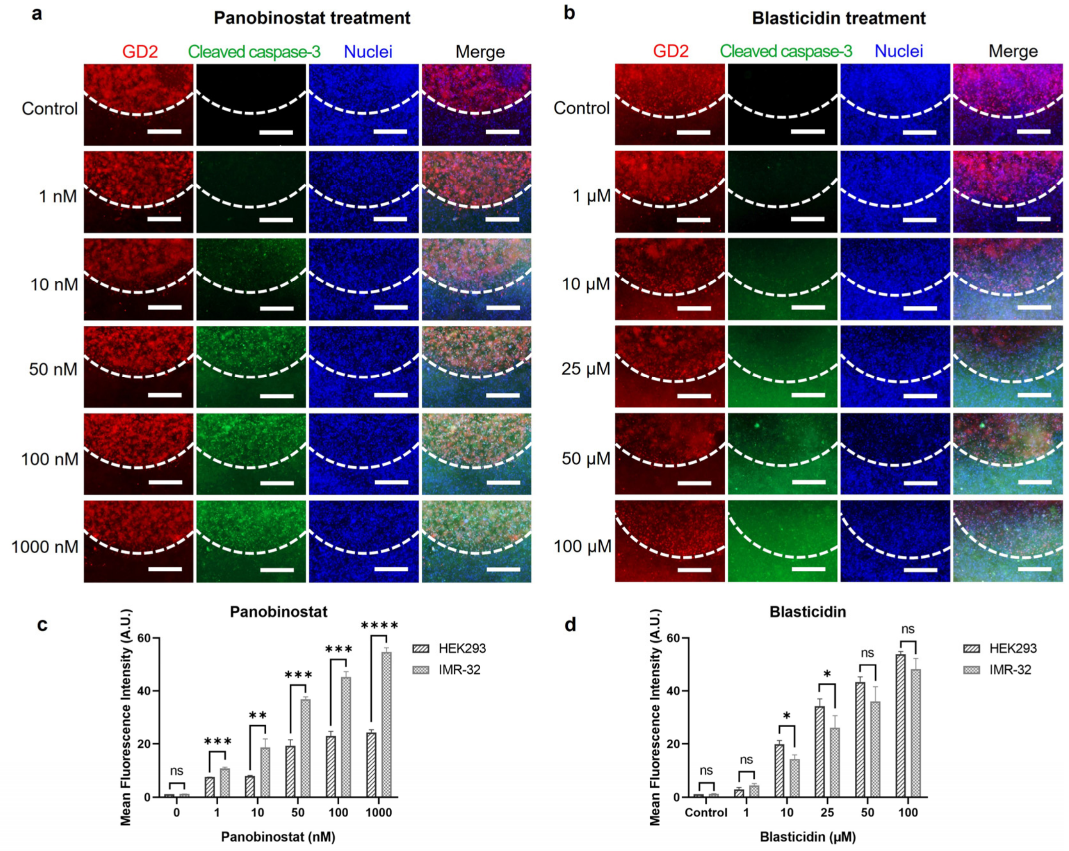

Figure S4. Apoptosis in cancer models after treatment with panobinostat or blasticidin. Cleaved caspase-3 was detected by immunofluorescence as a measure for the induction of apoptosis 24 h post treatment with panobinostat (a, c) or blasticidin (b, d). (a-b) Immunofluorescence images of cancer models after immunofluorescence staining. IMR-32 cells were labeled with a GD2 antibody. Cleaved caspase-3 was stained in green and nuclei were labelled with DAPI in blue. (c, d) Quantitative analysis of cleaved caspase-3 in non-cancerous environment and the cancer part of the models by mean fluorescence intensity. *p < 0.05, **p < 0.01, ***p < 0.001, ****p < 0.0001. Scale bar, 500 μm.

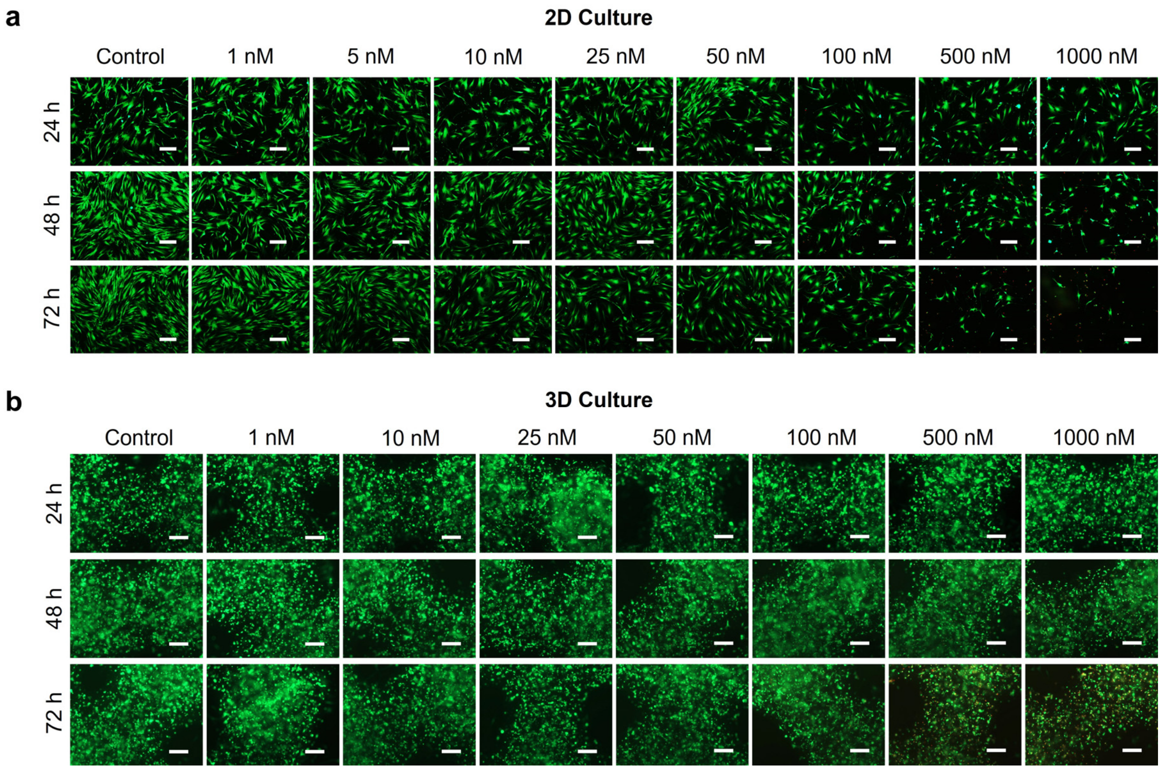

Figure S5. Cytotoxicity assays of human kidney fibroblast in 2D and 3D culture after treatment with panobinostat. Primary human kidney fibroblasts were either cultured in 2D (a) or bioprinted into 3D constructs (b). Following treatment with panobinostat for 24 h, 48 h and 72 h, cells were stained for the cytotoxicity assay. Living cells appear in green, and dead cells are in red. Scale bar, 250 μm.
